# Study protocol: identifying and delivering point-of-care information to improve care coordination

**DOI:** 10.1186/s13012-015-0335-9

**Published:** 2015-10-19

**Authors:** Sylvia J. Hysong, Xinxuan Che, Sallie J. Weaver, Laura A. Petersen

**Affiliations:** Center for Innovations in Quality, Effectiveness and Safety, Michael E. DeBakey Veterans Affairs Medical Center, Houston, TX USA; Department of Medicine—Health Services Research Section, Baylor College of Medicine, Houston, TX USA; Johns Hopkins University School of Medicine & Johns Hopkins Medicine Armstrong Institute for Patient Safety & Quality, Baltimore, MD USA; School of Psychology, Florida Institute of Technology, Melbourne, FL USA

## Abstract

**Background:**

The need for deliberately coordinated care is noted by many national-level organizations. The Department of Veterans Affairs (VA) recently transitioned primary care clinics nationwide into Patient Aligned Care Teams (PACTs) to provide more accessible, coordinated, comprehensive, and patient-centered care. To better serve this purpose, PACTs must be able to successfully sequence and route interdependent tasks to appropriate team members while also maintaining collective situational awareness (coordination).

Although conceptual frameworks of care coordination exist, few explicitly articulate core behavioral markers of coordination or the related information needs of team members attempting to synchronize complex care processes across time for a shared patient population. Given this gap, we partnered with a group of frontline primary care personnel at ambulatory care sites to identify the specific information needs of PACT members that will enable them to coordinate their efforts to provide effective, coordinated care. The study has three objectives: (1) development of measurable, prioritized point-of-care criteria for effective PACT coordination; (2) identifying the specific information needed at the point of care to optimize coordination; and (3) assessing the effect of adopting the aforementioned coordination standards on PACT clinicians’ coordination behaviors.

**Methods/design:**

The study consists of three phases. In phase 1, we will employ the Productivity Measurement and Enhancement System (ProMES), a structured approach to performance measure creation from industrial/organizational psychology, to develop coordination measures with a design team of 6–10 primary care personnel; in phase 2, we will conduct focus groups with the phase 1 design team to identify point-of-care information needs. Phase 3 is a two-arm field experiment (*n*_PACT_ = 28/arm); intervention arm PACTs will receive monthly feedback reports using the measures developed in phase 1 and attend brief monthly feedback sessions. Control arm PACTs will receive no intervention. PACTs will be followed prospectively for up to 1 year.

**Discussion:**

This project combines both action research and implementation science methods to address important gaps in the existing care coordination literature using a partnership-based research design. It will provide an evidence-based framework for care coordination by employing a structured methodology for a systematic approach to care coordination in PACT settings and identifying the information needs that produce the most successful coordination of care.

**Trial registration:**

ISRCTN15412521

**Electronic supplementary material:**

The online version of this article (doi:10.1186/s13012-015-0335-9) contains supplementary material, which is available to authorized users.

## Background

The importance of care coordination as a means to improving health care quality has been widely recognized. The Institute of Medicine has noted that “enhancing care coordination is essential to improving quality” [1]. In addition, a systematic review sponsored by the Agency for Healthcare Research and Quality (AHRQ) concerning care coordination strategies found that patients with chronic conditions such as congestive heart failure (CHF) and diabetes benefited most from care coordination interventions [1, 2]. Although numerous strategies designed to optimize coordination among multidisciplinary care teams have been proposed, high quality evidence and related insight into best practices for coordinating care remains underdeveloped.

The Department of Veterans Affairs (VA) recently completed implementation of Patient Aligned Care Teams (PACTs, known outside the VA as the Patient Centered Medical Home) [3], a team-based model of providing health care, into primary care clinics nationwide. PACTs aim to provide accessible, coordinated, comprehensive, and patient-centered care to address both the preventative and chronic care needs crucial for achieving population health. One of the most critical principles of PACT is care coordination.

Like any team-based model of work, PACTs work collectively on interdependent tasks to deliver evidence-based care that could not be accomplished as effectively by a single provider. For example, screening for colorectal cancer is a coordination-intensive PACT function. Previous work demonstrates that a primary care provider must go through a process involving as many as 25 steps and handoffs among up to eight different clinic personnel [4]. These complex processes are often prone to errors that affect quality, safety, value, and patient satisfaction. Effective coordination is central to addressing this problem. Research by Hysong and colleagues found that lack of policies and ambiguous roles and responsibilities (both essential components of coordination) are significant barriers to successful handoffs [5]. Additionally, their results highlighted timely, individualized, and customizable feedback and tracking tools as essential for both successful referral team coordination [5] and improved quality of care [6].

Nearly 30 years of research underscores coordination as one of the most critical functions of effective team-based work in healthcare [7]. Team members must be able to successfully sequence and route interdependent tasks, as well as collectively anticipate, time, and make sense of their work [7]. However, without necessary information and guidance on how to coordinate well, members in teams are less likely to be able to maximize their team efforts. This project explicitly seeks to identify the specific information needs of PACT members that will enable them to coordinate their efforts, resources, and processes in order to provide effective, coordinated care for all ambulatory care patients.

### Theoretical foundation: the Okhuysen and Bechky framework

Although considerable literature exists documenting the importance of care coordination [2, 8–11], an evidence-base for successful implementation of care coordination best practices remains scarce. This can be attributed to the lack of clear conceptual understanding of what coordination is and how best to achieve it. Although the AHRQ framework for coordinated care [12] provides a crucial starting point by enumerating specific coordination activities (e.g., facilitating transitions, assessing needs and goals, monitoring, and follow-up) as well as broad approaches to care (e.g., Health Information Technology-enabled coordination and a “health care home”), it fails to identify fundamental processes and mechanisms needed to coordinate successfully, i.e., collectively and effectively transition patients from primary to secondary care and vice versa [7, 13]. Seeking a stronger theoretical foundation, we used the Okhuysen and Bechky coordination mechanism framework [7], which is rooted in industrial/organizational (I/O) psychology (the scientific study of behavior in the workplace), as the theoretical foundation for this work.

Okhuysen and Bechky [7] propose an integrative framework explaining the mechanisms of coordination and the integrating conditions necessary to effectively achieve it. This model is a context-free representation of coordination and can easily be applied to the healthcare coordination context. The framework articulates five mechanisms underlying effective interdependent and collective performance, in other words, coordination (see Table 1).

**Table 1:** Essential mechanisms for effective coordination according to Okhuysen and Bechky’s [7] coordination framework

Coordination mechanism	Definition/example
Plans and rules	Explicit definitions of objectives, responsibilities, and resource allocations (e.g., Who is allowed to place a fecal occult blood test (FOBT) order?)
Objects and representations	Technologies, tools, and any device used to “create a common referent around which people interact, align their work, and create shared meaning” [7] (p. 474); for example, the use of templates to place a consult for a colonoscopy test.
Roles	Expectations of specific individuals. For example, which PACT member is supposed to follow-up with the patient once test results are available?
Routines	“Repeated patterns of behavior that are bound by rules and customs” [7] (p. 477). For example, when test results are completed, the ordering provider is notified.
Physical proximity among team members	For example, where are the ordering provider and the testing facilities located?

The five mechanisms enable three emergent conditions: (1) accountability (clarity over who is responsible for what), (2) predictability (knowing what tasks are involved and when they happen), and (3) common understanding (providing a shared perspective on the whole process and how individuals’ work fits within the whole). A successful integration of these three conditions allows team members to collectively accomplish interdependent tasks. Research in I/O psychology and management shows these mechanisms and conditions are associated with better coordination and subsequently better task performance [14, 15]. Thus, this conceptual framework provides a solid theoretical foundation for understanding the elements needed to help PACTs to more effectively coordinate care. Uriarte [16] provides an excellent, approachable summary of this theoretical framework.

As providers move from the traditional clinic structure to PACTs, they must adjust to several basic shifts in their work, including the shift from an individual provider focus to a team-based model of care [17]. Though tools and guidelines exist to aid in other transitions (e.g., from acute to chronic care and from single patient to population management), the transition from individual to team-based care has far less evidence-based support [18]. Other tools such as the Medical Home Builder® survey can help guide practices in setting up the right infrastructure for a team-based practice, and instruments such as the AHRQ’s Care Coordination Measures Atlas [2, 12] can provide a snapshot of broad-based care coordination; however, analogous guidance is not yet widely available for team-based care. This project will address this gap by identifying the information needs of PACT members that will allow them to coordinate their efforts, resources, and processes in order to provide effective, coordinated care in a team-based environment and will test the effectiveness of providing such information.

### Objectives

Guided by Okhuysen and Bechky’s coordination framework [7], “this project’s objective is to determine the point-of-care information needs of PACT members to successfully coordinate care and to identify the optimal mechanisms of delivering that information at the point-of- care*.*” We define point of care in the PACT context as any setting where a provider evaluates clinical information and makes a healthcare decision. This broad definition encompasses both traditional face-to-face encounters at clinical visits as well as non face-to-face interactions (e.g., telephone calls, secure messaging, or processing information from the electronic medical record) that are integral to PACT care delivery.

To accomplish our objective, we will employ the Productivity Measurement and Enhancement System (ProMES) to systematically identify organizational objectives and develop clear, accountable measures of coordination, which in turn will help identify care coordination needs in PACT settings. ProMES is a performance measure development method from I/O psychology rooted in 30 years of empirical and theoretical work in motivation, feedback, participation in decision making, and goal setting [19, 20]. ProMES uses specific criteria for evaluating measure quality, including several criteria used by the National Quality Forum (see Additional file 1). Via this methodology, we will accomplish the following:

1. Develop measurable criteria for effective coordination in PACTs, prioritized and weighted by contribution to overall quality of care.
2. Use the measurable criteria developed in Aim 1 to identify the specific information needed at the point of care to improve coordination and recommend point-of-care aids for delivering the needed information.
3. Assess the effect of adopting the aforementioned coordination criteria and feedback on PACT clinicians’ coordination behaviors. Our hypothesis:
  *H*_1_—Coordination behaviors at sites adopting measurable criteria and feedback will be significantly more effective than coordination behaviors at control sites.

## Methods/design

As this protocol is intended to describe the details of a partnership-based research project, we followed recently published recommendations for reporting such work [21].

### Project description and study design

This project consists of three phases: (1) developing measurable, prioritized point-of-care criteria for effective PACT coordination, to be accomplished using ProMES (detailed below); (2) identifying the specific information needed at the point of care to improve coordination and recommend point-of-care aids for delivering the needed information, to be accomplished via focus groups with the phase 1 participants; and (3) assessing the effect of adopting the aforementioned coordination standards and point-of-care aids on PACT clinicians’ coordination behaviors.

**Fig. 1:**
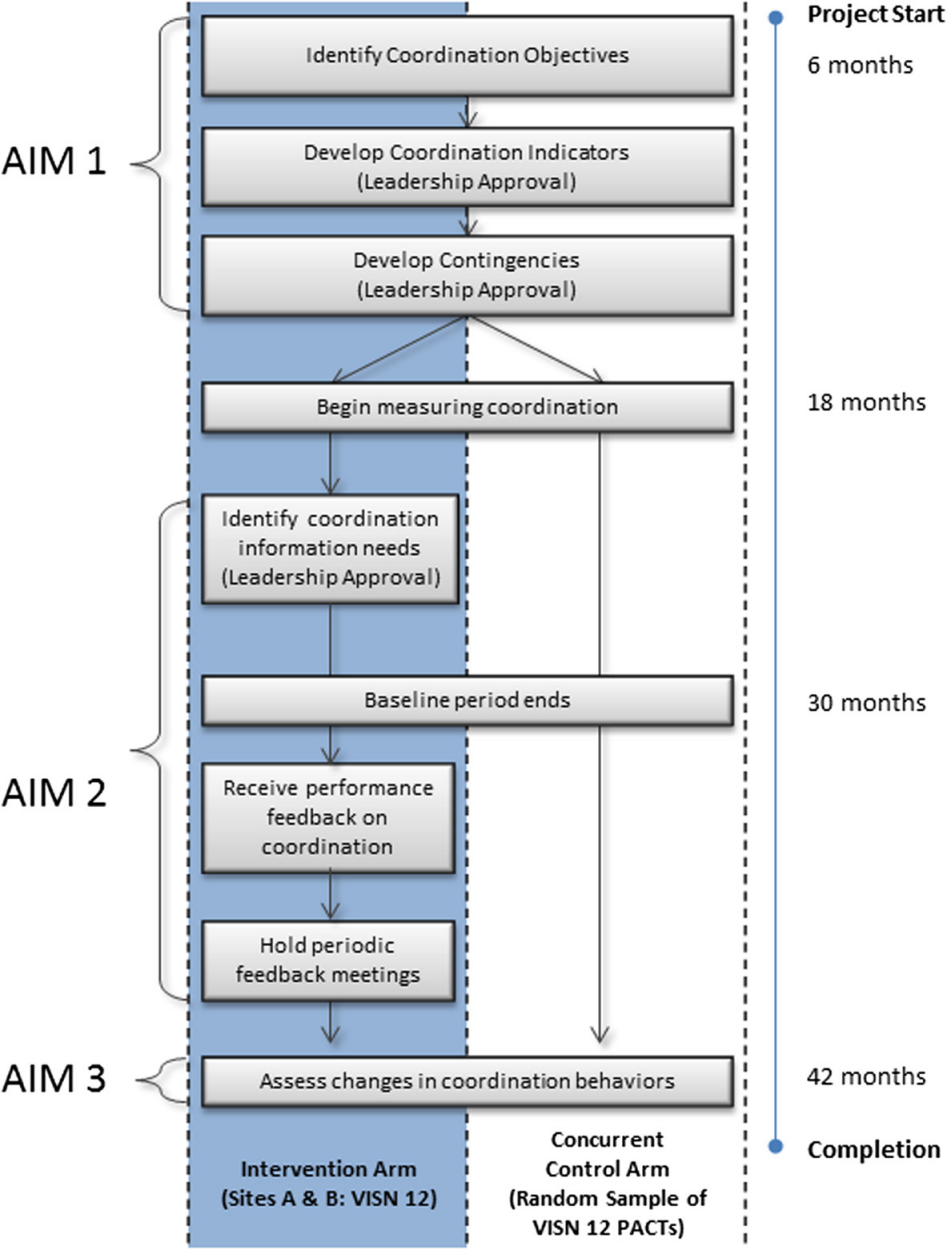
Study design

Phase 3 consists of a trial with two arms: (1) a concurrent control arm drawn randomly from PACTs which will be monitored passively and (2) a measurement and feedback arm (two sites from a single regional network), which will be involved in designing the coordination measures and identifying the point-of-care aids and will receive periodic feedback on their coordination performance. The design of the three phases of the study is summarized in the flow chart in Fig. 1. Each phase is discussed below in detail.

### Partnership approach

#### Research team

The issues and research questions addressed by this project require a multidisciplinary team. Experts in the field of I/O psychology (SJH, SJW, XC), public health (TF), internal medicine (LAP), data management and computer programming (MK), and statistics (AA) bring together complementary expertise to provide the necessary knowledge, skills, and experience to successfully carry out the study.

#### Operational partners

This project will be carried out in close partnership with the VA Great Lakes Healthcare system (known within VA as Veterans Integrated Service Network (VISN) 12 or Network 12), which has seven VA medical centers and over 30 VA clinics in areas of north-western Indiana, northern Illinois, Wisconsin, and the Upper Peninsula of Michigan. During the years, Network 12 has been dedicated to excel in providing services to America’s veterans by being innovative, productive, responsive, and accountable. In this project, Network 12 provides protected time for their personnel to participate in all three phases of the study as well as access to related clinical data. The partnership enables us to implement the study design in a targeted setting with the capacity to evaluate both scientific and operational results.

#### Relationship between research team and operational partners

Dr. Laura Petersen, co-principal investigator of this study, has had a longstanding relationship with Network 12, having conducted numerous performance measurement projects under contract with Network 12 for over a decade. This allows for an equal partnership between the research team and Network 12: the research team providing the scientific and methodological expertise as well as protected research effort, and Network 12 providing study sites, data, and protected operational time for clinical staff to participate in the work. In addition, to ensure engagement of key stakeholders with the project, an Executive Steering committee is in place to provide guidance and assistance for engagement with project sites throughout the project. This steering committee is composed of leaders and key stakeholders both at participating sites and the VISN office.

## Phase 1: develop measurable, prioritized point-of-care criteria for effective PACT coordination

### Overview of ProMES methodology

A common way to help develop new tools and products such as the point-of-care aids, we propose to identify in phase 2, is the use of focus groups to identify user needs [3, 22]. One drawback of the traditional focus group approach, however, is that it can easily result in haphazard and unsystematic identification of barriers, facilitators, and solutions, particularly in the hands of an inexperienced facilitator. With a complex topic such as coordination, unsystematic responses are even more likely. The complexities of this topic demand a structured, systematic process to walk users logically and comprehensively through all of the relevant components of coordination. Once PACT members have a clear picture of the criteria for successful coordination, identifying coordination information needs will be more feasible. Thus, developing measures and criteria for effective coordination not only teaches PACT members what effective coordination should look like but also will help identify coordination information requirements in patient care. To accomplish this aim, we will use ProMES [20, 23] as the primary method of data collection and identification of point-of-care information needs.

ProMES is a comprehensive productivity improvement approach firmly grounded in motivational theory [24, 25] and performance measurement. It utilizes diagnostic measurement to optimize the effectiveness and performance of people in complex organizations. ProMES is designed with implementation in mind: incumbents of a given work unit, supervisors, and upper level management will engage in the development of measures for capturing each important aspect of their daily work (in this case coordination). Through a facilitated process, these measures are defined, weighted, and prioritized in order to create indicators of both overall effectiveness and specific aspects of daily work. Moreover, ProMES helps address all three of Okhuysen and Bechky’s integrating conditions of coordination. The process of identifying objectives helps clarify expectations (plans and rules), which helps improve shared understanding among PACT members; the process of developing indicators and contingencies helps clarify roles and routines (thereby improving predictability and accountability) by making expectations clear, thus requiring team members to think about what they need to perform to standard.

Previous research in intensive care unit and mental health care settings has shown that successful use of ProMES can improve efficiency and quality of care. For example, in studies conducted with a German mental health hospital the ProMES methodology was utilized across organizational levels for the top management team, samples of nurses, and three samples of technicians. Results indicated that overall productivity scores for participating units were, on average, 0.78 standard deviations higher after implementation [23].

### Participants

The first step in ProMES is to form a “design team” that will actively participate in the development process. Ideally, design teams consist of 6–8 representatives from an intact work group or department (preferably of varying roles), the work group’s supervisor, and 1–2 facilitators. For this study, we will form a team composed of key informants from each job title in the PACT teamlet and extended team (provider, nurse/case manager, LVN, clerk, dietician, pharmacist, social worker). In addition, we will recruit a representative from each specialty that treats the conditions of interest (oncology, cardiology), as well as a Primary Care Team Leader (who will also fulfill the supervisor role in the traditional ProMES design team configuration). Finally, the design team will have two facilitators, one based in Houston and one based in the Network 12 area. The facilitators will be independent of the process, rather than representatives of any of the aforementioned parties. Two experts in ProMES will train the facilitators in ProMES with specific emphasis on the objectives of this study, including promoting the project to colleagues at their facility. We will work with our existing Network 12 contacts to identify suitable individuals to serve on the design team.

In this project, we are also using ProMES to develop a separate intervention: identification of point-of-care information needs. These steps stand to materially impact existing workflows in the clinic; to maximize implementation success, buy-in is necessary from more than simply the design team members and their leadership. Consequently, we will create an advisory team composed of a similar job title and site distribution as the design team, plus relevant stakeholders as needed, including key leaders. The advisory team will provide feedback to the design team at key points in the process and will help champion the resulting aids to facilitate implementation at their home sites. The design team facilitator will also facilitate advisory team activities, to help maintain some organizational memory.

### Procedures

The design team will participate in structured meetings to identify coordination objectives (i.e., what is accomplished by coordinating care) and coordination indicators (i.e., how do we know we are achieving our coordination goals?), and to assess the relative importance and value of each indicator, referred to as “contingencies”. These meetings aim to achieve a concrete set of standards and measures designed to gauge how well PACT teams are performing the act of coordinating. Although there is a recommended total number of hours of face time for each step, the specific meeting schedule will be tailored to meet the participants’ needs.

### Step 1: identify care coordination objectives

The design team will be led by facilitators in a 1-day exercise to identify care coordination objectives (see Table 2 for examples). Objectives will be defined by design team members in the context of PACTs coordinating care for the three conditions of interest. For each clinical condition, the design team is charged with answering, “what is the PACT trying to accomplish when coordinating care?” Trained facilitators will guide the design team to arrive at three to six objectives that meet six recommended criteria ([23]; see also Additional file 1). Objectives will be compared against these criteria to ensure their utility in subsequent steps.

**Table 2:** Sample potential coordination objectives and indicators resulting from ProMES methodology

Objective	Indicator
1. Optimize balance of quality vs. length of life given patient preferences	1. Score on patient preferences/satisfaction survey
	2. Length of life compared to algorithm based on stages of disease
2. Ensure timely screening, delivery, and follow-up of care	3. Mean number of days between date provider orders test and date clerk schedules appointment with the patient
	4. Percent of patients eligible for screening tests who receive them in the specified period.
	5. Percent of patients eligible for diagnostic tests who receive them in the specified period.
3. Ensure care is evidence-based and comprehensive by providing the right expertise mix to care for patient	6. Percent of provider type match per patient problem, i.e., the correct type of provider should be working on a patient for every problem on the patient problem list

Sample objectives and indicators are from a brief pilot ProMES session conducted for care coordination specific to cancer care.

### Step 2: develop care coordination indicators

#### Generating indicators

After identifying the PACT’s care coordination objectives for the conditions of interest, the design team will develop indicators. For each objective the design team answers the following question: “How would you show that the PACT is meeting the stated objective?” To accomplish this, the design team will ideally convene in a series of weekly 1–2 h facilitator-led meetings for a total of approximately 25 h of face time. In these meetings, they will identify a set of performance markers that capture the extent to which the coordination objectives are being achieved. This schedule helps limit fatigue and cognitive bias and allows for reflection while still keeping the process sufficiently fresh to make continued progress. It also provides the opportunity to test the feasibility of collecting marker data and consider barriers (e.g., intrusiveness). We will work with our Network 12 partners to develop a schedule that is feasible for design team members while still maintaining the scientific integrity of the process.

#### Ensuring indicator quality

Each indicator must meet multiple criteria regarding its validity, comprehensiveness, impact, feasibility, and usability (see Additional file 1). An indicator information form will be used to ensure the criteria are addressed and document all relevant information for each indicator (see Additional file 2). The resulting indicators will be used as the measures needed to assess change in care coordination (i.e., phase 3).

#### Advisory team review

Once indicators are drafted, the objectives and indicators will be presented to the advisory team for review and feedback. This step is essential for checking accuracy and completeness of the system, ensuring alignment with leadership objectives, and securing buy-in from outside the design team. To accomplish this, the incumbent design team members (not the facilitator, though he/she will be in attendance) will present the objectives and indicators to the advisory team in a 1-h meeting.

### Step 3: prioritize indicators by developing contingencies

**Fig. 2:**
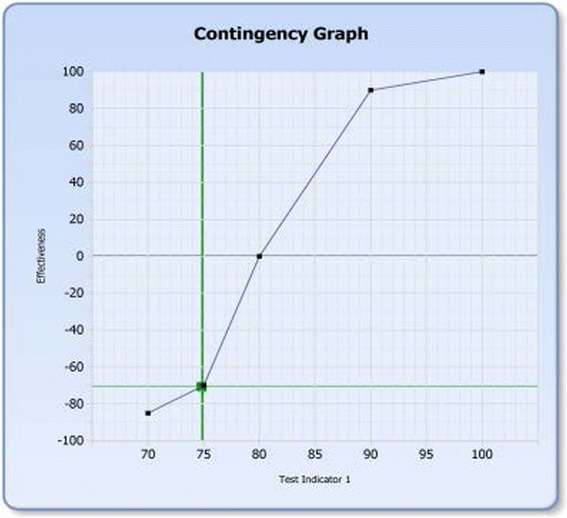
Sample contingency graph. Note: This graph is used to help translating the score of the indicator to effectiveness on the overall coordination. Zero on the vertical axis represents the expected level of the indicator. One hundred on the vertical axis represents the maximum level of the most important indicator. Minus 100 represents the minimum level of the No. 1 minimum indicator if performing poorly on this indicator effect overall coordination as much as the most important indicator. Then assign max scores to each indicator relatively to the most important indicator and the min scores to each indicator relatively to the No. 1 minimum indicator

In the ProMES methodology, contingencies refer to what *level* of performance is acceptable or how much a given level of improvement is valued. Contingencies are represented graphically as a function that shows how much a given performance level on a given indicator contribute to overall effectiveness (in this case PACT coordination effectiveness; see Fig. 2). A contingency function is generated for each indicator. By relating each indicator to overall coordination effectiveness, they are put on the same measuring scale, ranging from −100 to +100. Thus, the various indicators can be directly compared, prioritized, and combined into a single measure. Most importantly, it reflects an explicit statement of relative priority among the different elements of coordination and defines levels of coordination-related performance that are expected and valued by the PACT and the facility.

#### Creating contingencies

The design team will convene for a total of 6–8 h to develop a contingency function for each indicator. The design team must identify the maximum and minimum possible levels of performance, the minimum expected performance level, and scale the various levels of performance to a common metric of effectiveness for each indicator. Pritchard and colleagues explain this protocol in detail [23].

#### Leadership approval and implementation planning

Once the list of contingencies has been completed to the satisfaction of the group, the incumbent design team members (not the facilitator, though he/she will be in attendance) will present them in a 1–2 h meeting with senior leadership to obtain formal approval of the contingencies.

### Data analysis

No data analysis will occur during developmental phase 1.

## Phase 2: identify the specific information needed at the point of care to improve coordination and recommend point-of-care aids for delivering the needed information

### Design

For this aim, the design and advisory teams will participate in structured meetings in a format similar to ProMES to identify point-of-care coordination information needs. In addition, using the measures created in phase 1, we will begin collecting baseline coordination data at the sites intended to be studied in phase 3.

### Participants

The participants for this aim will be the members of the design and advisory teams described in phase 1.

### Procedures

#### Begin measuring coordination

Upon completion of phase 1, we will begin securing the necessary permissions to obtain the data required for generating the indicators developed during phase 1. Subsequently, measurement of PACT coordination will begin at all four sites, using the measures developed in that aim. The period between completion of phase 1 and identification of point-of-care aids will serve as a baseline; during this period, we will provide no feedback, only passively monitor coordination.

#### Develop point-of-care aid recommendations

The final development step will involve crafting a document recommending specific point-of-care aids by which PACT members could receive prioritized point-of-care information. The prioritized list of coordination objectives and performance indicators developed in phase 1 provide PACT members with critical guidance: they know what the objectives are in coordinating care, what is expected of them, which aspects of coordination are most important, and what level of performance is considered acceptable. The team now has what it needs to identify its coordination information needs. For each objective and its respective indicators, the design team will review the performance indicators and contingency functions to answer the question, “What coordination information does each PACT member need to accomplish these objectives to the levels of performance described in these indicators and contingency functions?” The design team will be guided by the Okhuysen and Bechky model, and they will identify information needs with the goal of maximizing accountability, predictability, and common understanding. Once the list is generated, the design team can use the indicator contingencies to prioritize the information needs. To accomplish this, the design team will convene for a total of 6–8 h. Focus groups meetings will be recorded and field notes taken in order to accurately capture the design team’s recommendations.

We will hold two to four meetings with the design team to elicit the requirement characteristics of the point-of-care aids. The discussion will be guided both by Okhuysen and Bechky’s model as well as literature on best practices for feedback delivery [26, 27].

#### Leadership approval

The research team will compile the results from the multiple meetings into a single set of findings. These findings will be presented to the advisory team for their review and feedback. The design team will then refine the point-of-care aid ideas based on the advisory team’s feedback. The design team and research team together will then present the finalized point-of-care aid recommendations to leadership for approval.

## Phase 3: assess the effect of adopting the aforementioned coordination standards and point-of-care aids on PACT clinicians’ coordination behaviors

### Design and site selection

Our study will take place at four facilities within Network 12 with a high degree of alignment with PACT. Within this subset, we will select sites based on geography (rural vs. urban) and size (CBOC vs. VAMC) to ensure maximum variation; we will approach sites uninvolved in other projects from this CREATE to ensure an uncontaminated study design.

### Participants

For this phase, we will recruit members of all available PACTs at the four Network 12 study sites. Given our site selection criteria, we estimate each study arm will have an average of approximately 152 members clustered into 28 PACTs. For purposes of informed consent, we will treat all PACT members at a site as study subjects; we are aware that not all PACT members may consent to participate; however, our intervention is an intent-to-treat design, and the unit of analysis is the PACT, not the individual PACT member (our outcome of interest, coordination, is by definition a collective construct). Consequently, as long as at least the provider of a given PACT consents, we will include that PACT’s data in our analyses, taking into consideration the limitations of that PACT’s data.

Optimal design software developed by Raudenbush and colleagues [28] was used to calculate the estimated effect size for varying levels of power for a multilevel cluster randomized trial with repeated measures. With a two-tailed alpha of 0.05 and 6 sites with an average of 10 PACTs per site, an intra-class correlation coefficient for sites of 0.02 (estimated from prior VA data), and approximately 12 repeated assessments, we have 80 % power to detect a medium-to-large effect size (*δ* = 1.13) and 87 % power to detect a large effect (*δ* = 1.25). Pritchard and colleagues’ [19] meta-analysis examining the effectiveness of ProMES as improving productivity found a large overall effect size (*δ* = 1.44); accounting for moderators, effect sizes were as high as 2.21. Thus, we should be able to adequately detect improvements using the proposed number of PACTs.

### Procedures

#### Begin measuring coordination

Assessment of change requires a baseline; consequently, we will use the time period between completion of phase 1 and completion of phase 2 to passively monitor coordination at all study sites.

#### Identify point-of-care aids

The performance period will begin upon final approval by leadership of the point-of-care aid recommendations document.

#### Provide periodic and feedback

We will provide monthly audit and feedback reports on the PACTs’ coordination performance (as measured by the indicators developed in phase 1) to all PACTs in the intervention arm. We will use standardized, ProMES-based audit and feedback reports as a starting point [29]. We will work with our Network 12 partners to adapt reports to their needs based on their suggestions and what research has shown to maximize feedback effectiveness [26, 27].

These reports will be utilized in feedback meetings between the PACT Teams and the PACT Team Leader to identify barriers and facilitators to productivity and discuss strategies to implement needed change. PACT Team Leaders will be trained on how to run these feedback meetings. Training will include interpretation of the feedback report, as well as instruction on delivering feedback in a team setting and discussing the results with the PACTs. Previous research suggests monthly feedback meetings constitute an appropriate time interval for providing face-to-face feedback of this sort [19]. We will therefore suggest monthly feedback meetings, adjusting this frequency interval based on the feedback needs of the PACT and availability of indicator data.

#### Adjust system as necessary

Three months after the performance period begins, the design and advisory teams will review the point-of-care aids, providing suggestions for adjustments. The design and research teams will collect suggestions and convene for two 2-h meetings to make adjustments as needed. Coordination will then be monitored from that point forward using the revised point-of-care aids.

### Measures

#### Coordination

Coordination will be assessed using the measures developed in phase 1. In addition, the contingency process enables us to form an overall care coordination composite, defined as the simple sum of the indicators [23]. No weighting or additional transformations are necessary, as nonlinearity and complexities are accounted for in the indicators’ contingencies (i.e., the transformation from raw score to an effectiveness scale).

#### Care coordination

In addition to the ProMES-developed care coordination measures, we will also monitor existing care coordination measures in the PACT Compass (VA’s dashboard for reporting a variety of quality, safety, and value measures related to PACTs) for each study site. As of this writing, these measures consist of the following: ER/Urgent Care Utilization Rate by Panel, Inpatient Admission Rate, 2-day contact post-discharge ratio, and 7-day contact post-discharge ratio. With both standard measures of care coordination and the ProMES-developed measures of coordination in hand, it will be possible to discern the extent to which the values of these existing measures of care coordination are contingent upon coordination behaviors among PACT members. We will also be able to compare agreement between measures of coordination and standard process measures currently in use.

### Data analysis

For each objective, we will create composite score of coordination effectiveness, calculated per ProMES recommendations, as the simple sum of all the indicators relevant to that objective. The unit of analysis will be the PACT. We will compare across arms the improvement in coordination effectiveness score over time (baseline vs. performance periods) using growth curve modeling. Coordination effectiveness would be the dependent variable; time point and study arm would be level 1 and 2 predictors, respectively. Each will be modeled separately. A significant, positive main effect of time and study arm will indicate differential improvement across arms over time; an intervention arm slope that is significantly steeper than the control arm indicates support for our hypothesis. In addition to our planned hypothesis test, we also have the opportunity to investigate the extent to which performance on existing care coordination measures is a function of PACT coordination effectiveness. We will conduct separate analyses for each care coordination measure in the PACT Compass. For each, we will test a growth curve model with Compass measure as the dependent variable and time, study arm, and the coordination effectiveness composites as predictors. A significant, positive main effect of coordination effectiveness after accounting for time and study arm would indicate support for the hypothesis that better PACT coordination behaviors is associated with better care coordination outcomes as currently measured by the PACT Compass.

### Trial status

Phase 1 of this study, measure development, is nearly complete. Both design and advisory teams were formed to develop and evaluate the objectives, indicators, and contingencies. These development meetings are complete; we are now finalizing the specific data sources for the indicators developed, which will be used to prepare the feedback reports in phase 3. The core study team is currently in phase 2 of the study and preparing for phase 3. We have modified our control arm sampling strategy from all available PACTs at two VISN 12 sites to a random sample of PACTs from anywhere in VISN 12 to minimize contamination.

## Discussion

### Potential challenges and limitations

This project faces several potential challenges and limitations. First, the research team and the participant team are geographically dispersed. This creates challenges for participatory action research, training, and for effective communication. Virtual meetings and training strategies will be critical. Second, the availability of clinical participants can be limited in many circumstances. In these cases, the design team and advisory team meetings may not have diverse representation of every role in the PACTs. Third, given the size of Network 12 and the availability of the clinicians, it is impossible for us to have representations from all the PACTs. This may limit the generalizability of the coordination measures identified and deemed acceptable by the teams.

### Contributions to science and practice

This project will materially contribute to both the science and practice of care coordination in several ways. Because of its partnership-based design, this project is in some respects a special case of a type 1 hybrid implementation trial: it evaluates the *effectiveness* of the feedback intervention tested in phase 3 but incorporates elements in all three phases that allow us to adapt and tailor the *implementation* of the intervention to our partners’ needs. Upon completion of the project, our partners will have practical, feasible, and prioritized behavioral measures of care coordination in PACT settings, which in conjunction with regular feedback, can help PACTs pinpoint areas for improvement. These measures have the added benefit of potential for adoption beyond the borders of our operational partner. In addition, PACTs will have tangible aids at the point of care to help them coordinate more smoothly, and thereby, provide better quality of care. This project also contributes to science by identifying elements of coordination that are important regardless of clinical condition or disease, thereby helping to unify the currently fragmented literature on care coordination and team-based care. Finally, the methodology we propose to use to develop the aforementioned measures, ProMES, will be used for the first time not simply as the performance measure development tool for which it is originally intended but rather as an implementation intervention to aid in identifying the interventions needed to improve coordination. By using ProMES to systematically identify the indicators of good coordination, point-of-care coordination information needs become self-evident. This innovative use of ProMES also marks a contribution to both implementation science and management science more broadly.

## Additional files

Additional file 1:
**Measurement Criteria For ProMES Objectives and Performance Indicators [** 22 **].** (PDF 15 kb) Additional file 2:
**ProMES indicator information form.** (PDF 15 kb)

## Abbreviations

CHF: congestive heart failure
PACTs: Patient Aligned Care Teams (known outside the VA as the Patient Centered Medical Home)
ProMES: Productivity Measurement and Enhancement System
VA: Department of Veterans Affairs
Network 12: Veterans Integrated Service Network 12

## References

[1] Committee on Quality of Health Care in America (2006). Rewarding Provider Performance: Aligning Incentives in Medicare.

[2] McDonald KM, Sundaram V, Bravata DM, Lewis R, Lin N, Kraft S, McKinnon M, Paguntalan H, Owens DK (2007). Closing the quality gap: a critical analysis of quality improvement strategies. Vol 7 -- Care Coordination. Volume 04(07)-005.

[3] U.S. Department of Veterans Affairs. VA Primary Care Services. Patient-centered medical home model concept paper. Washington, DC: U.S. Department of Veterans Affairs Website. http://www.va.gov/health/services/PrimaryCare/docs/pcmh_ConceptPaper.doc. Accessed October 16, 2015.

[4] Hysong SJ. Organizational Correlates of Outpatient Performance Trends in VAMCs. Annu Career Dev Meet Veterans Adm Heal Serv Res Dev Serv. Washington, DC; 2011

[5] Hysong SJ, Esquivel A, Sittig DF, Paul LA, Espadas D, Singh S, et al. Toward successful coordination of electronic health record based-referrals: a qualitative analysis. Implement Sci. 2011;6. 10.1186/1748-5908-6-84 PMC3199858 21794109

[6] Hysong SJ, Best RG, Pugh JA (2006). Audit and feedback and clinical practice guideline adherence: making feedback actionable. Implement Sci.

[7] Okhuysen GA, Bechky BA (2009). Coordination in organizations: an integrative perspective. Acad Manag Ann.

[8] Stille CJ, Jerant A, Bell D, Meltzer D, Elmore JG (2005). Coordinating care across diseases, settings, and clinicians: a key role for the generalist in practice. Ann Intern Med.

[9] Forrest CB, Glade GB, Baker AE, Bocian A, von Schrader S, Starfield B (2000). Coordination of specialty referrals and physician satisfaction with referral care. Arch Pediatr Adolesc Med.

[10] Berendsen AJ, de Jong GM, Meyboom-de Jong B, Dekker JH, Schuling J (2009). Transition of care: experiences and preferences of patients across the primary/secondary interface—a qualitative study. BMC Heal Serv Res.

[11] Bal R, Mastboom F, Spiers HP, Rutten H (2007). The product and process of referral: optimizing general practitioner-medical specialist interaction through information technology. Int J Med Inform.

[12] McDonald KM, Schultz E, Albin L, Pineda N, Lonhart J, Sundaram V, et al. Care Coordination Measures Atlas. Agency for Healthcare Research and Quality (AHRQ); 2010. http://www.ahrq.gov/qual/careatlas/index.html

[13] Salas E, Sims DE, Burke CS (2005). Is there a “Big Five” in teamwork?. Small Gr Res.

[14] Marks MA, Panzer FJ (2004). The influence of team monitoring on team processes and performance. Hum Perform.

[15] Stout RJ, Salas E, Carson R (1994). Individual task proficiency and team process behavior: what’s important for team functioning?. Mil Psychol.

[16] Uriarte J. Working well together: how coordination happens. [Video file]. 2015. Retrieved from https://www.youtube.com/watch?v=B85K_uklrTo.

[17] Glasgow RE, Orleans CT, Wagner EH (2001). Does the chronic care model serve also as a template for improving prevention?. Milbank Q.

[18] Taplin SH, Weaver S, Salas E, Chollette V, Edwards HM, Bruinooge SS, et al. Reviewing Cancer Care Team Effectiveness. J Oncol Pract. 2015:JOP.2014.003350–. 10.1200/JOP.2014.003350 PMC4438110 25873056

[19] Pritchard RD, Harrell MM, Diaz-Granados D, Guzman MJ (2008). The productivity measurement and enhancement system: a meta-analysis. J Appl Psychol.

[20] Pritchard RD, Kleinbeck U, Quast HH, Thierry H, Haecker H (1990). Enhancing work motivation through productivity measurement and feedback. Work Motivation.

[21] Hysong SJ, Woodard L, Garvin JH, Murawsky J, Petersen LA (2014). Publishing protocols for partnered research. J Gen Intern Med.

[22] Barbour R (2008). Doing focus groups.

[23] Pritchard RD, Weaver SJ, Ashwood EL (2011). Evidence-based productivity improvement: a practical guide to the productivity measurement and enhancement system.

[24] Naylor JC, Pritchard RD, Ilgen DR (1980). A theory of behavior in organizations.

[25] Pritchard RD, Ashwood EL (2007). Managing motivation: a manager’s guide to diagnosing and improving motivation.

[26] Hysong SJ (2009). Meta-analysis: audit & feedback features impact effectiveness on care quality. Med Care.

[27] Kluger AN, DeNisi A (1996). The effects of feedback interventions on performance: a historical review, a meta-analysis, and a preliminary feedback intervention theory. Psychol Bull.

[28] Raudenbush S (2006). Optimal design for multilevel longitudinal research.

[29] Kleingeld AD, Van Tuijl H, Algera JA (2004). Participation in the design of performance management systems: a quasi-experimental field study. J Organ Behav.

