# Supplementary material for: Study protocol: identifying and delivering point-of-care information to improve care coordination
Source: Implement Sci. 2015 Oct 19;10:145. doi: 10.1186/s13012-015-0335-9 (PMC4613788; doi:10.1186/s13012-015-0335-9)
Supplement: Additional file 1: — Measurement Criteria For ProMES Objectives and Performance Indicators [ 22 ]. (PDF 15 kb) [file 13012_2015_335_MOESM1_ESM.pdf]

Additional file 1 – Measurement Criteria For ProMES Objectives and Performance Indicators[22]

1. CRITERIA FOR PROMES OBJECTIVES

Below are the criteria used to evaluate the quality of performance objectives according to the ProMES model. Objectives that meet these criteria are important prerequisites for identifying and developing appropriate performance measures.

- Objectives should be stated in clear terms
- Objectives should be designed so that if exactly that objective was accomplished, the organization would benefit
- The set of objectives must cover all important aspects of the work (in our case, all important aspects of coordination)
- Objectives must be consistent with the objectives of the broader organization
- Leadership must be committed to each objective
- The number of objectives should be manageable, normally 3 to 8.

2. CRITERIA FOR PROMES INDICATORS

Below are the criteria used to evaluate the quality of a performance measure according to the ProMES model. For consistency with more health-care specific model, they are organized according to the criteria used by the National Quality Forum to evaluate clinical measures.

1. Validity/Reliability
   - Indicators must validly measure the objective
   - Indicators must be largely under the control of unit personnel
   - The information provided by the indicator must be neither too general nor too specific.
2. Comprehensiveness (not part of NQF criteria)
   - All important aspects of each objective must be covered by the set of indicators
3. Impact (Value)
   - Indicators must be consistent with the objectives of the broader organization
   - Indicators should be designed so that if the indicator was maximized (i.e., perfect score), the organization would benefit (value – similar to NQF's Impact)
4. Feasibility
   - Leadership must be committed to each indicator
   - Accurate indicator data must be cost effective to collect
5. Usability
   - Indicators must be understandable and meaningful to unit personnel
   - It must be possible to provide information on the indicator in a timely manner
