# Supplementary material for: Study protocol: identifying and delivering point-of-care information to improve care coordination
Source: Implement Sci. 2015 Oct 19;10:145. doi: 10.1186/s13012-015-0335-9 (PMC4613788; doi:10.1186/s13012-015-0335-9)
Supplement: Additional file 2: — ProMES indicator information form. (PDF 15 kb) [file 13012_2015_335_MOESM2_ESM.pdf]

Additional file 2 – ProMES indicator information form

Facilitator Name: PACT Lead:

Obj	
Name	
Number	

Indicator 1

Name:			
Rationale:			
Indicator Calculation:			
Data Source:			
Assignments:			
Data Collection:		Data Entry:	

Indicator 2

Name:			
Rationale:			
Indicator Calculation:			
Data Source:			
Designates:			
Data Collection:		Data Entry:	
